# Prednisolone Alters Endometrial Decidual Cells and Affects Decidual-Trophoblast Interactions

**DOI:** 10.3389/fcell.2021.647496

**Published:** 2021-04-09

**Authors:** Eliza Grbac, Teresa So, Swati Varshney, Nicholas Williamson, Evdokia Dimitriadis, Ellen Menkhorst

**Affiliations:** ^1^Department of Obstetrics and Gynaecology, The University of Melbourne, Parkville, VIC, Australia; ^2^Gynaecology Research Centre, Royal Women’s Hospital, Parkville, VIC, Australia; ^3^Melbourne Mass Spectrometry and Proteomics Facility, Bio21 Molecular Science and Biotechnology, Parkville, VIC, Australia; ^4^Centre for Reproductive Health, Hudson Institute of Medical Research, Clayton, VIC, Australia; ^5^Department of Anatomy and Developmental Biology, Monash University, Clayton, VIC, Australia

**Keywords:** prednisolone, decidualization, trophoblast, recurrent pregnancy loss, preeclampsia

## Abstract

Poor pregnancy outcomes such as recurrent pregnancy loss (RPL) and preeclampsia are associated with impaired decidualization and abnormal trophoblast invasion. Emerging evidence suggests that use of corticosteroids, including prednisolone affects fertility by altering uterine function and may be associated with preeclampsia incidence. In this study, using primary and gestational-age appropriate tissue, we aimed to define the effect of prednisolone on human endometrial stromal fibroblast (hESF) decidualization and determine whether hESF decidualization in the presence of prednisolone would alter hESF regulation of trophoblast function. We found that prednisolone treatment reduced hESF cytokine expression (*IL6*, *IL11*, *IL18, LIF*, and *LIFR*) but had no effect on hESF expression or secretion of the classic markers of decidualization [prolactin (PRL) and IGFBP1]. Using proteomics we determined that prednisolone altered decidualized hESF protein production, enriching hESF proteins associated with acetylation and mitrochondria. Conditioned media from hESF decidualized in the presence of prednisolone significantly enhanced trophoblast outgrowth and trophoblast mRNA expression of cell motility gene *PLCG1* and reduced trophoblast production of *PGF*. Prednisolone treatment during the menstrual cycle and 1st trimester of pregnancy might alter decidual interactions with other cells, including invasive trophoblast.

## Introduction

To prepare for embryo implantation and pregnancy, uterine endometrial stromal fibroblast [human(h)ESF] differentiate or “decidualize” in response to progesterone to become decidual cells (Evans et al., 2016). Decidualization is initiated immediately post-ovulation under the control of progesterone and involves the reprogramming of hESF such that different genes are expressed at different stages of differentiation (Popovici et al., 2000). During implantation extravillous trophoblast (EVT) invades into the decidualized endometrium (decidua) and upper third of the myometrium (Lunghi et al., 2007). The decidua produces factors which regulate trophoblast invasion (Dimitriadis et al., 2005; Lunghi et al., 2007; Burton et al., 2010; Menkhorst et al., 2012, 2015, 2019; Pollheimer et al., 2018) and protect the conceptus from the maternal immune system and oxidative stress (Evans et al., 2016; Okada et al., 2018). Poor pregnancy outcomes including recurrent pregnancy loss (RPL) and preeclampsia are associated with impaired decidualization (Founds et al., 2009; Salker et al., 2010; Dimitriadis et al., 2020).

Prednisolone is a corticosteroid which classically acts *via* the glucocorticoid receptor (GR) and has anti-inflammatory and immuno-modulatory effects (Frolkis et al., 2010). Prednisolone administration during the menstrual cycle and early pregnancy may affect endometrial stromal and trophoblast cells: GR are present in the glandular epithelium and stromal cells of the endometrium and 1st trimester decidua (Henderson et al., 2003) as well as trophoblast cells of the 1st trimester placenta (Yang et al., 2016; Kisanga et al., 2018). Murine models suggest that prednisolone or other corticosteroid administration during early pregnancy affects fertility and pregnancy outcome *via* actions on the uterus (Matejevic et al., 1995; Li et al., 2018; Kieffer et al., 2020), however, the precise impact of these drugs on endometrial cells specifically is unknown.

Prednisolone is used an off-label therapy for RPL to reduce uterine Natural Killer (uNK) cell numbers (Dimitriadis et al., 2020). The reported efficacy of prednisolone at preventing miscarriage in women with a history of idiopathic miscarriage is highly variable between studies (Tang et al., 2013; Gomaa et al., 2014; Dan et al., 2015; Cooper et al., 2019) however, the most recent systematic review and meta-analysis found that prednisolone administration did not improve miscarriage rates or pregnancy outcome (Woon et al., 2020). Concerningly, there is emerging evidence linking corticosteroid use to preeclampsia incidence in women (Boyd et al., 2015; Bandoli et al., 2017) and dexamethasone treatment in pregnant rats induces the development of PE features (Zhang et al., 2016, 2018), however, most studies investigating the role of prednisolone in RPL are not sufficiently powered to identify rare pregnancy outcomes such as preeclampsia.

We hypothesized that prednisolone treatment may have off-target actions on endometrial stromal fibroblasts, affecting decidualization, decidual regulation of trophoblast function and ultimately the formation of a healthy placenta. In this study, using primary and gestational-age appropriate tissue, we aimed to define the effect of prednisolone on hESF decidualization and determine whether hESF decidualized in the presence of prednisolone would differently regulate trophoblast function.

## Materials and Methods

This study was conducted under approvals from The Royal Women’s Hospital and Monash Health Human Research and Ethics Committees (#90317B, #06014C, and #03066B). Written and informed consent was obtained from each patient before surgery. All experiments were performed in accordance with the NHMRC guidelines for ethical conduct in human research.

Endometrial biopsies were collected by dilatation and curettage from women (*n* = 15 women; age 36.4 ± 1.3 years; range 29–46 years). Women were fertile (*n* = 2/15), primary infertile (*n* = 3/15; unable to conceive for ≥12 months), secondary infertile (*n* = 9/15; unable to conceive for >6–12 months but who have had a previous successful pregnancy), or unknown fertility (*n* = 1/15; have not attempted to conceive). 4/15 women had polyps, 1/15 had endometriosis, 1/15 had PCOS, 2/15 had menorrhagia and the reminder (7/15) had no obvious endometrial pathology. The women had no hormonal treatment for ≥3 months before tissue collection, however, 2/15 were prescribed prednisolone.

Products of conception were collected from first trimester pregnancies (*n* = 9; amenorrhea 5–13 weeks) following elective termination of pregnancy by evacuation for psychosocial reasons.

### Culture Conditions

All cells were cultured at 37°C in a 5% CO_2_ humidified culture incubator. hESF were maintained in DMEM/F12 (Gibco, Thermo Fisher Scientific, Inc.) plus 10% charcoal stripped Fetal Bovine serum (FBS; Gibco, Thermo Fisher Scientific, Inc.) and 1% antibiotics (penicillin, streptomycin, amphotericin B; Gibco, Thermo Fisher Scientific, Inc.). Isolated EVTs were maintained in DMEM/F12 containing 10% heat-inactivated FBS (Gibco, Thermo Fisher Scientific, Inc.) and 1% antibiotics.

### Decidualization

hESF were isolated as previously described by collagenase digestion and filtration (Menkhorst et al., 2017) which results in a 97% pure stromal cell culture (Dimitriadis et al., 2002). hESF were decidualized as previously described (Menkhorst et al., 2017). Briefly, hESF were treated with estradiol (E, 10^–8^ M; Sigma) alone or E plus medroxyprogesterone acetate (MPA, 10^–7^ M; Sigma) in DMEM/F12 containing 2% charcoal stripped FBS and 1% antibiotics for up to 14 days. The media was refreshed every 2–3 days, on a Monday, Wednesday, and Friday. 9/15 cultures (eight secondary infertile, one fertile) were frozen after isolation and subsequently thawed for decidualization, proteomics and trophoblast experiments; 6/15 (two fertile, three primary infertile, and one unknown) were decidualized without being frozen and thawed for decidualization and trophoblast experiments.

### Prednisolone Treatment

**FIGURE 1:**
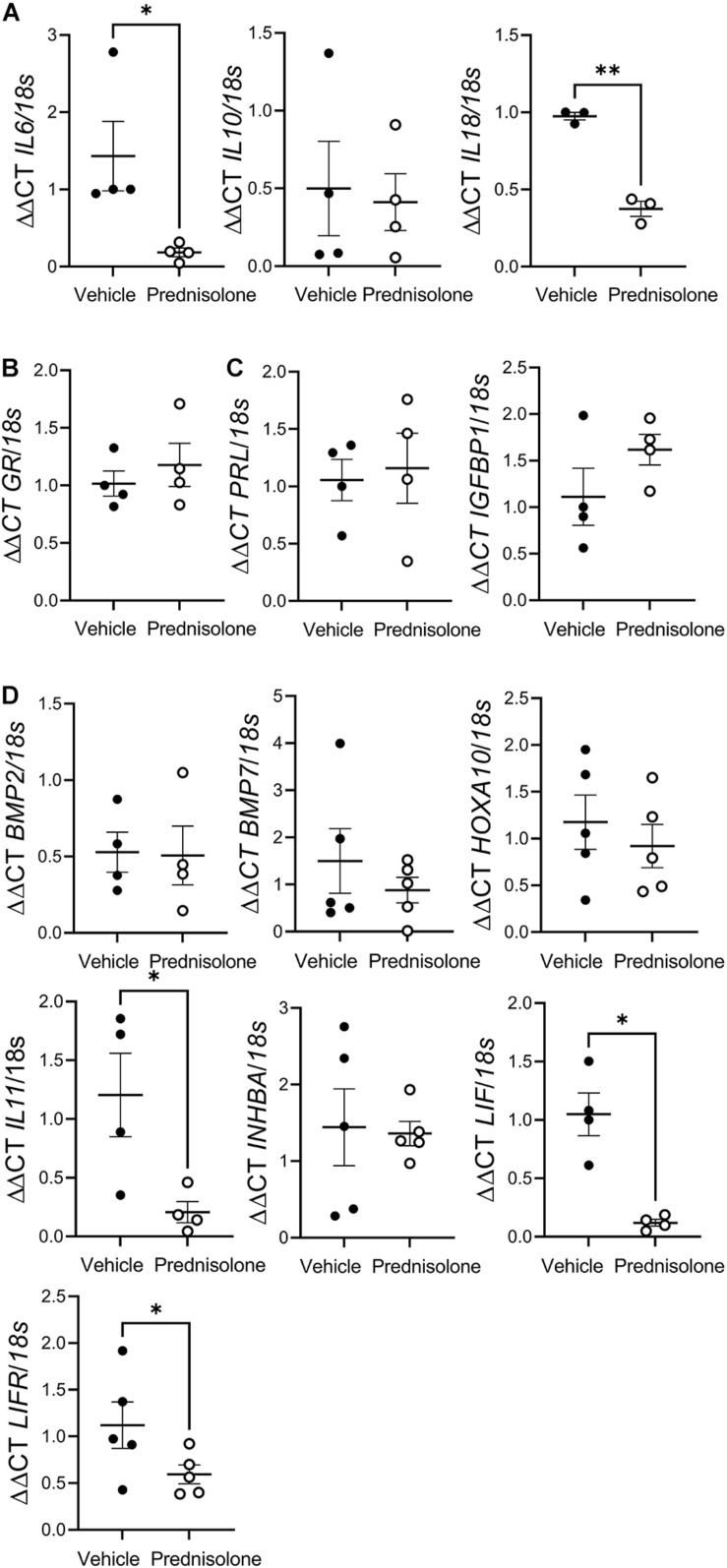
Prednisolone suppressedpro-inflammatory cytokine production by human endometrial stromalfibroblast (hESF). Prednisolone treatment: **(A)** inhibitedhESF pro-inflammatory cytokine [*interleukin* (IL) *6*, and *IL18*] gene expression, but had no effect on *IL10* gene expression, paired *t*-test, *n* = 3-4/group; **(B)** had no effect on hESF *glucocorticoid receptor* (*GR*) gene expression, paired *t*-test, *n* = 4/group; **(C)** had no effect on classic decidualization makers *prolactin* (*PRL*) or *insulin-like growth factor binding protein (IGFBP)1* expression; **(D)** had no effect on decidualization genes *bone morphogenic protein (BMP)2*, *BMP7*, *homeobox A (HOXA)10* or *inhibin*β*A (INHBA)*, but inhibited *IL11*, *leukemia inhibitory factor (LIF)* and *LIF receptor (LIFR)* gene expression, paired *t*-test, *n* = 4/group. Data presented as mean ± SEM; *P < 0.05.

To determine the effect of prednisolone on hESF gene expression, non-decidualized hESF (*n* = 5 biological replicates) were treated with prednisolone (0.5 μg/ml; Aspen Pharmacare; vehicle control DMSO) for 16 h. To determine the effect of prednisolone treatment during decidualization, hESF (*n* = 11 biological replicates) undergoing *in vitro* decidualization as described above were treated with prednisolone (0.5 μg/ml) for 12–13 days. On days 9/10 or 12/13 media that had been incubated with cells for 48 h (conditioned media, CM; added to cells either days 7/8 or 10/11) was collected for prolactin (PRL) or insulin-like growth factor binding protein 1 (IGFBP1) ELISA. The day of collection varied as decidualization treatments were started as soon as the hESF were confluent and media was only changed on Monday, Wednesday, and Friday. On day 13 hESF were washed twice with PBS and cells were cultured for a further 24 h in decidualization treatment (E + MPA) only (prednisolone washout). Cells and CM collected from decidualized hESF on day 14 was pooled and used for trophoblast/outgrowth treatments and day 14 hESF cells (*n* = 3) were used for gene expression and proteomics analyses. The dose of prednisolone was determined from concentration in plasma following a single 20 mg dose (Wilson et al., 1977) and subsequent trial of various doses (0.05, 0.5, and 5 μg/ml) *in vitro*. We found 0.5 μg/ml prednisolone effectively suppressed hESF pro-inflammatory cytokine production (Figure 1A).

### Treatment of Trophoblast With hESF Conditioned Media

#### Trophoblast Outgrowth

Trophoblast outgrowth from villous tips (*n* = 6 biological replicates) was quantified as previously described (Winship et al., 2015; Menkhorst et al., 2019) with slight modification: villous tips were seeded on to neat growth-factor reduced Matrigel^TM^ (Corning) instead of collagen. After 48 h of culture outgrowing villous tips were treated with day 14 hESF CM (FC 50%) for 72 h. The tips were photographed at 48 and 120 h and area of outgrowth quantified using ImageJ at 120 h (normalized to outgrowth at 48 h).

#### EVT Gene Expression

Trophoblast were isolated as previously described (Menkhorst et al., 2012) and cultured on growth factor reduced Matrigel^TM^ diluted 1:5 in DMEM/F12 to promote differentiation toward the EVT phenotype. EVTs (*n* = 3 biological replicates) were treated with neat day 14 hESF CM for 16 h before RNA isolation for gene expression analysis.

### Prolactin and IGFBP1 ELISA

Prolactin and IGFBP1 secretion by decidualized hESF (days 9/10 and 12/13) was quantified by ELISA of hESF CM as per the manufacturer’s instructions (DuoSet kits #DY682 and #DY871, R&D systems). Briefly, capture antibody was diluted in phosphate buffered saline and used to coat a 96 well microplate overnight at room temperature (RT; 100 μL/well). The following morning the capture antibody was aspirated before the plate was washed three times with wash buffer before non-specific antibody binding was blocked by incubation with reagent diluent (300 μL) for 1 h at RT. The plate was again washed before 100 μL standards or hESF CM was added to the plate and incubated for 2 h at RT. Each standard or sample was assayed in duplicate technical replicates. hESF CM was assayed neat for the PRL ELISA and diluted 1:2 in reagent diluent for the IGFBP1 ELISA. Following a further wash step 100 μl detection antibody was incubated for 2 h at RT. The plate was again washed before 100 μl Streptavidin-HRP complex was incubated for 20 min at RT. The plate was washed a final time before 100 μl of substrate solution was added to each well (incubated for 20 min at RT) and finally minutes 50 μl of Stop solution added to each well. The optical density of each well was immediately determined using a microplate reader (Biostrategy Spectramax PLUS Plate Reader) set to 450 nm.

### Gene Expression

RNA extraction and quantitative RT-PCR was performed as previously described (Menkhorst et al., 2020) using Tri Reagent (Sigma-Aldrich) or the RNeasy mini kit (QIAGEN), Superscript III First-Strand Synthesis System (Thermo-Fisher) and Power SYBR Green master mix (Applied Biosystems) on the Veriti 7 fast block real-time qPCR system (Applied Biosystems). A template-free negative control in the presence of primers and RNase-free water only was added for each run and each sample assayed in triplicate technical replicates. Primer sequences are shown in Supplementary Table 1; primers were obtained from Sigma-Aldrich. The qPCR protocol was as follows: 95°C for 10 min and 40 cycles of 95°C for 15 s followed by 60°C for 1 min. Relative expression levels were calculated using comparative cycle threshold method (ΔΔCT) as outlined in the manufacturer’s user manual.

*PCR Array:* To determine the potential mechanisms by which prednisolone-treated hESF induced trophoblast outgrowth we used a QIAGEN Cell Motility Array (PAHS-1282A) as per the manufacturer’s instructions on EVT treated with hESF CM. RNA was pooled from *n* = 2 tissues for the array.

### Mass Spectrometry

Decidualized hESF (*n* = 3 biological replicates) cellular proteins following decidualization including treatment with 0.5 μg/ml prednisolone or vehicle control were identified using mass spectrometry. Day 14 cells were lysed and homogenized in ice-cold universal lysis buffer as previously described (Menkhorst et al., 2012).

Full details are provided in the Supplementary Material. Briefly, 3 μg total cellular protein quantified using BCA assay (Pierce) was used for Solid-Phase Protein Preparation followed by LC-MS/MS as previously described (Dagley et al., 2019; Hughes et al., 2019). The analysis of the samples was based on the label-free quantification (LFQ) intensities. Initial analyses and visualization of proteomics data was performed using LFQ-Analyst (Shah et al., 2020). The data was statistically evaluated using Perseus software (version 1.6.7.0). The mass spectrometry proteomics data have been deposited to the ProteomeXchange Consortium *via* the PRIDE (Perez-Riverol et al., 2019) partner repository with the dataset identifier PXD020543. Assessment of protein function enrichment was performed using DAVID Bioinformatics Resources 6.8^1^ (Huang Da et al., 2009b, a), selecting *Homo sapiens* as the reference species.

### Statistical Analysis

GraphPad Prism 9.02 was used for all statistical analysis. Paired*t*-tests and repeated measures ANOVA were used. All data is presented as mean ± SEM. *p* < 0.05 was considered statistically significant.

## Results

### Prednisolone Regulated hESF Cytokine Production

To confirm that prednisolone was active in hESF we determined whether prednisolone altered mRNA expression of cytokines known to be regulated by prednisolone. Prednisolone treatment of non-decidualized hESF significantly inhibited mRNA expression of the pro-inflammatory cytokines *interleukin* (*IL) 6* (7.8-fold), and *IL18* (2.6-fold), but had no effect on *IL10* (Figure 1A) or *GR* expression (Figure 1B) compared to control.

### Prednisolone Had No Effect on the Classical Markers of hESF Decidualization

We determined whether prednisolone could directly regulate genes associated with decidualization: prednisolone had no effect on *PRL, IGFBP1* (Figure 1C), *bone morphogenic protein (BMP) 2, BMP7, homeobox A (HOXA) 10*, or *inhibin*β*A (INHBA)* production, but significantly inhibited *IL11* (sixfold), *Leukemia inhibitory factor* (*LIF;* eightfold), and *LIF receptor* (*LIFR;* twofold) mRNA expression (Figure 1D).

**FIGURE 2:**
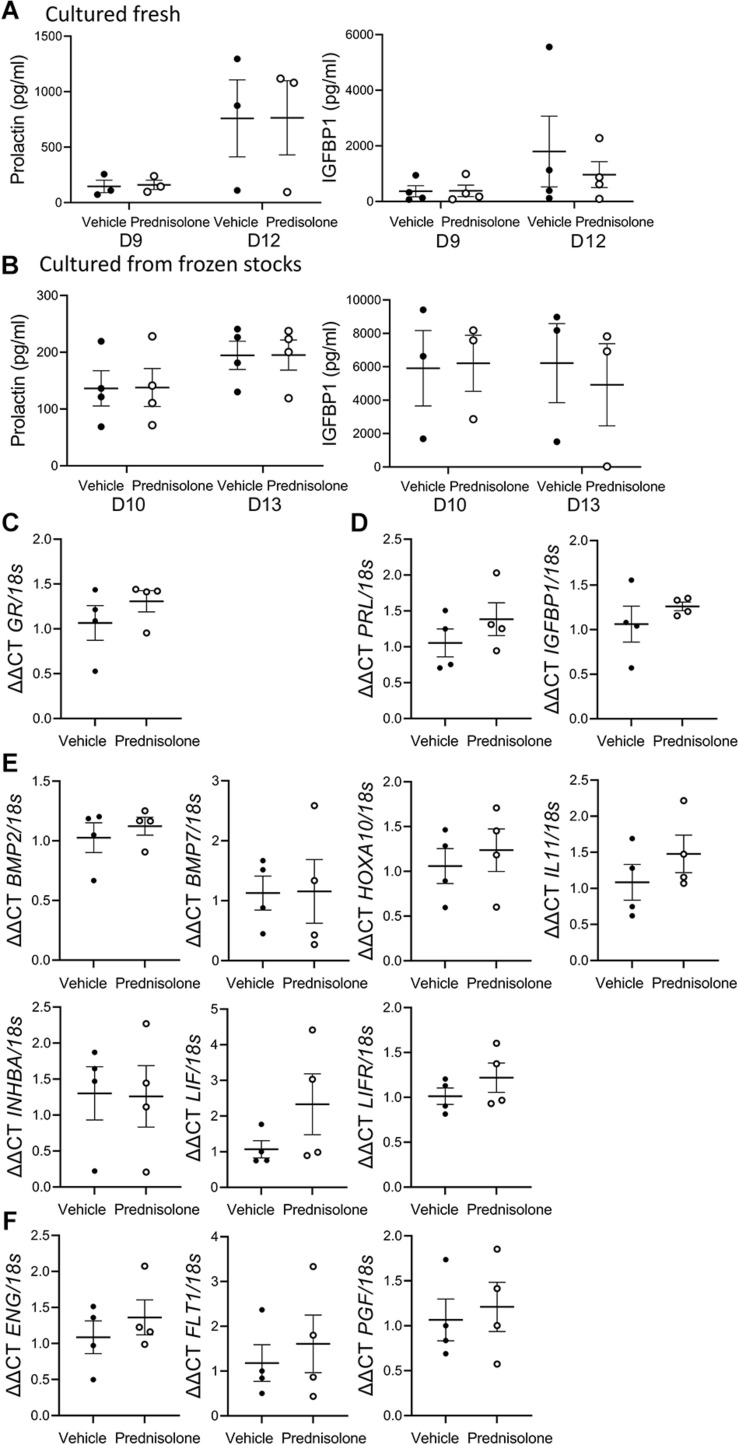
Prednisolone had no effect on classic decidualization markergene expression or secretion by decidualized human endometrial stromal fibroblast (hESF). Prednisolone treatment: **(A,B)** had no effect on decidualized hESF secretion of PRL or IGFBP1 from hESF **(A)** cultured fresh or **(B)** cultured from frozen stocks, repeated measures ANOVA; *n* = 3-4/group; **(C–F)** had no effect on decidualized hESF production of **(C)** *GR*; **(D)** classic decidualization markers *PRL* or *IGFBP1*; **(E)** decidualization genes *BMP2, BMP7, HOXA10, IL11, INHBA, LIF or LIFR*; **(F)** preeclampsia-associated genes *endoglin* (*ENG), vascular endothelial growth factor receptor (FLT1)* or *placental-like growth factor (PGF)*, paired *t*-test, *n* = 4/group; Data presented as mean ± SEM; *P < 0.05.

Long-term treatment of hESF with estrogen plus MPA induced decidualization as demonstrated by detectable PRL and IGFBP1 secretion (Figures 2A,B; E alone treatment showed undetectable PRL and IGFBP1 at Day 9/10 and 12/13, data not shown). Addition of 0.5 μg/ml prednisolone to the decidualization treatment showed no significant effect on PRL or IGFBP1 secretion in hESF cultured fresh (Figure 2A; 3/4 primary infertile, 1/4 unknown fertility) or frozen down before seeding for decidualization treatments (Figure 2B; 3/4 secondary infertile, 1/4 fertile).

Decidualized hESF gene expression following long-term treatment with hESF was examined 24 h after prednisolone withdrawal (day 14). There was no effect of prednisolone on decidualized hESF *GR* (Figure 2C), *PRL*, *IGFBP1* (Figure 2D), *BMP2, BMP7, HOXA10, IL11, INHBA, LIF, LIFR* (Figure 2E), *endoglin (ENG), vascular endothelial growth factor receptor (FLT1)*, or *placental-like growth factor (PGF)* production (Figure 2F).

### Prednisolone Altered Decidualized hESF Protein Production

We performed proteomics on hESF cellular protein following *in vitro* decidualization in the presence of prednisolone (0.5 μg/ml) or vehicle control (DMSO) to identify decidualized hESF proteins regulated by prednisolone. We identified 2,254 individual proteins with >2 peptides in control decidualized hESF by mass spectrometry. We quantitated the production of 1,824 individual proteins between control and prednisolone-treated hESF.

**FIGURE 3:**
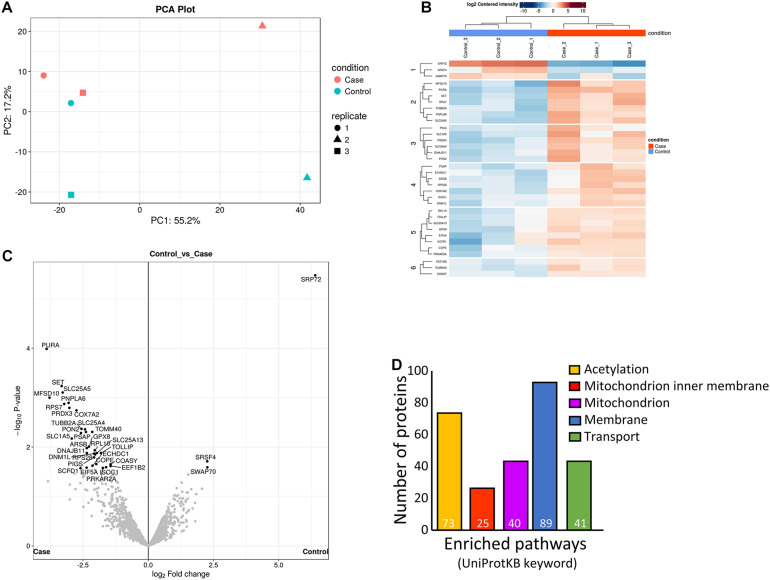
Analysis of differently regulated decidualized human endometrial stromal fibroblast (hESF) proteins following *in vitro* decidualization in the presence of prednisolone. **(A)** Principal components analysis. **(B)** Heat-map. **(C)** Volcano plot. **(D)** Enriched pathways. Individual numbers of proteins identified is indicated in white at the base of each bar. Case: prednisolone treated hESF; Control: vehicle control treated hESF.

Prednisolone treatment substantially altered decidualized hESF protein production (Figures 3A,B). 176 proteins showed significant fold-changes following prednisolone treatment (Figure 3C), including one down-regulated (Signal recognition particle subunit SRP72) and 175 up-regulated (Supplementary Table 2). Functional clustering analysis (DAVID) identified that hESF decidualized in the presence of prednisolone had enrichment of proteins associated with acetylation (3.4-fold), mitochondrion inner membrane (14.6-fold), mitochondrion (5.7-fold), membrane (1.9-fold), and transport (3.3-fold) (Figure 3D and Supplementary Table 3).

### Trophoblast Outgrowth Was Enhanced by hESF Decidualized in Presence of Prednisolone

**FIGURE 4:**
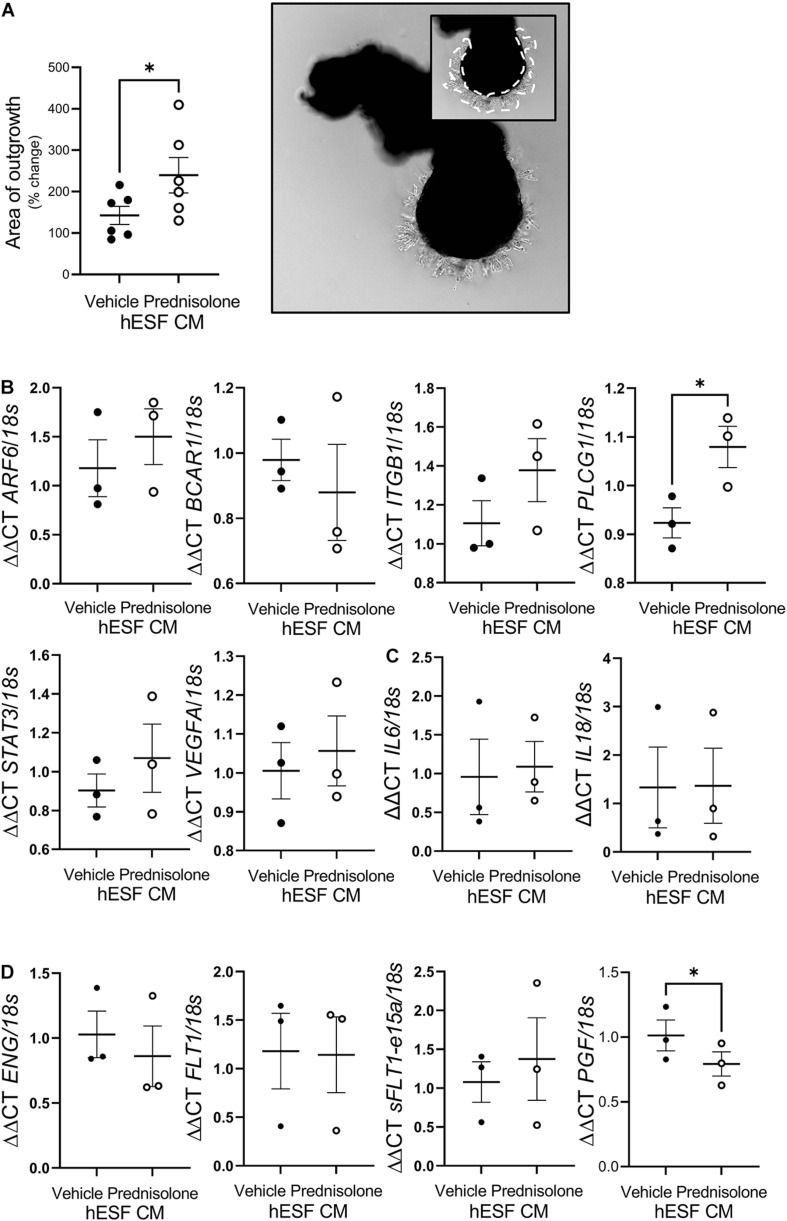
Prednisolone altered human endometrial stromal fibroblast (hESF) regulation of trophoblast function. **(A)** Extravillous trophoblast (EVT) outgrowth was significantly enhanced by conditioned media (CM) from hESF decidualized in the presence of prednisolone. Image shows representative outgrowth from villous tip. Insert shows area of outgrowth highlighted by dotted line. Paired *t*-test, *n* = 6. **(B)** EVT *PLCG1* expression was significantly increased by treatment with CM from hESF decidualized in the presence of prednisolone. Paired *t*-test, *n* = 3/group. **(C)** EVT *IL6* and *IL18* expression was not altered by treatment with CM from hESF decidualized in the presence of prednisolone. Paired *t*-test, *n* = 3/group. **(D)** EVT *PGF* expression was significantly inhibited by treatment with CM from hESF decidualized in the presence of prednisolone. Paired *t*-test, *n* = 3/group. Data presented as mean ± SEM; *P < 0.05.

To determine whether decidualization in the presence of prednisolone would impact decidual-trophoblast interactions we determined the effect of hESF CM on EVT outgrowth. EVT outgrowth was significantly enhanced following treatment with CM from hESF decidualized in the presence of prednisolone (1.7-fold, Figure 4A) compared to hESF decidualized in the presence of the vehicle control. The potential mechanism by which CM from hESF decidualized in the presence of prednisolone enhanced EVT outgrowth was investigated by assessing EVT gene expression following treatment with decidualized hESF CM (16 h) using a cell motility array (Supplementary Table 4). Genes highly altered on the array were validated by qPCR (Figure 4B). *Phospholipase C, gamma 1* (*PLCG1*) was significantly increased in EVT exposed to prednisolone-treated decidualized hESF CM (1.2-fold, Figure 3B). To further investigate the effect of CM from hESF decidualized in the presence of prednisolone, we assessed EVT expression of genes associated with inflammation and preeclampsia (Figures 4C,D). *Placental-like Growth Factor* (*PGF*) was significantly decreased in EVT exposed to prednisolone-treated decidualized hESF CM (1.3-fold, Figure 4D).

## Discussion

This is the first study to investigate the effect of prednisolone treatment on decidualization and decidual-trophoblast interactions. Prednisolone treatment during *in vitro* decidualization did not alter production of the classic decidualization markers PRL or IGFBP1 but altered hESF cytokine gene expression and decidualized hESF cellular protein. Intriguingly, trophoblast-decidual interactions were altered following hESF decidualization in the presence of prednisolone: we found prednisolone treatment enhanced trophoblast outgrowth, elevated EVT *PLCG1* production and reduced EVT *PGF* production.

To our knowledge the direct effect of prednisolone on decidualization in women has never been investigated. Although prednisolone has been shown to downregulate *GR* production in HeLa cells (Shimojo et al., 1995) here we saw no effect on *GR* production in non-decidualized or decidualized hESF. Our data suggests that prednisolone suppresses hESF pro-inflammatory cytokine production as has previously been shown in other cell types (Karagiannidis et al., 2004; Andersson et al., 2005; Shmarina et al., 2017). It is interesting that prednisolone suppressed hESF production of *IL11*, *LIF*, and *LIFR* which we previously showed enhanced progesterone-induced decidualization (Dimitriadis et al., 2003; Shyua et al., 2011). Inhibition of *IL11* expression by methylprednisolone has previously been show in bronchial epithelium (Chakir et al., 2003), but there is no previous investigation of the effect of prednisolone on *LIF* production. As IL11 and LIF are only two of many pathways altered during decidualization (Gellersen and Brosens, 2014; Evans et al., 2016) it is unsurprising that suppression of IL11 and LIF did not lead to altered production of PRL or IGFBP1, however, dysregulation of these factors may still impact hESF decidualization. It must be noted that the absolute levels of PRL and IGFBP1 secretion were different between hESF cultured fresh vs. those which were frozen before thawing for culture experiments. As most of the fresh hESF were from women with primary infertility and most of the frozen hESF were from women with secondary infertility this difference could also reflect the clinical characteristics of the women. Regardless, prednisolone had no effect on PRL or IGFBP1 production by hESF.

Since we found no effect of prednisolone treatment on gene expression or secretion of the classical markers of decidualization, we performed mass spectrometry to identify whether hESF proteins were altered by prednisolone treatment during decidualization. Of the 176 hESF proteins significantly regulated by prednisolone, 27 had previously been identified in decidua, including factors which promote decidualization [including GNA11/GNAQ (De Oliveira et al., 2019), CDK6 (Tan et al., 2002), and SCRIB (Whitby et al., 2018; Yuan et al., 2019)], or which are upregulated during decidualization [including CTTN (Paule et al., 2011), CTNNA1 (Patterson et al., 2017), SPTLC2 (Ding et al., 2018), and ALDH1A1 (Tomari et al., 2020)]. Decidualization itself is associated with substantial post-translational modification (Díaz-Gimeno et al., 2014) and here we found that prednisolone stimulated the production of proteins associated with acetylation, however, the effect of altered acetylation in decidualized hESF is unknown. Prednisolone treatment also altered hESF proteins associated with mitochondria, including increased production of factors associated with ATP generation and transport (e.g., UQCRC2, VDAC1/2; ATP5 synthases; NDUF enzymes; SLC25A mitochondrial carrier proteins). Again, the precise effect of altered hESF mitochondrial function is unknown. It is likely that proteins regulated by prednisolone will alter decidual cell interactions with other cells in the uterus, including trophoblast as demonstrated here.

We previously observed that CM from decidualized hESF enhanced trophoblast outgrowth when compared to non-decidualized hESF (Menkhorst et al., 2019). Despite prednisolone having no effect on classic markers of decidualization we observed that CM from hESF decidualized in the presence of prednisolone enhanced trophoblast outgrowth, suggesting that prednisolone altered hESF release of factors which regulate trophoblast function. Future studies are required to elucidate how prednisolone alters hESF CM and thus decidual regulation of trophoblast invasion, however, the data presented here suggests hESF CM may alter trophoblast motility genes including *PLCG1*. PLCG1 has not previously been identified in trophoblast or the placenta, however, it has well established roles in tumor metastasis where it promotes cell invasion (Kunze et al., 2014; Jang et al., 2018; Tang et al., 2019), potentially *via* its interactions with MMP2 (Zhang et al., 2019) or EGFR signaling (Jang et al., 2018). The precise effect that increased *PLCG1* production by EVTs would have on trophoblast invasion and whether hESF CM regulates other trophoblast functions including viability or proliferation remains to be experimentally determined.

There is emerging evidence linking corticosteroid use to preeclampsia incidence in women (Bandoli et al., 2017) and the development of PE features in rodents (Zhang et al., 2016, 2018). The Danish National Cohort study identified corticosteroid medication use for inflammatory bowel disease had a strong and significant association with preeclampsia (Boyd et al., 2015). Diseases for which prednisolone is a common treatment are also conditions with elevated risk of preeclampsia, including Antiphospholipid syndrome (APS) (when prescribed in addition to aspirin and heparin) (Empson and Al, 2005), chronic kidney disease (10-fold increased risk of preeclampsia) (Wiles et al., 2020) and systemic lupus erythematosus (14% increased risk of preeclampsia) (Chen et al., 2019), however, the contribution of prednisolone vs. the effect of the disease itself to preeclampsia risk has not been established.

The contribution of decidual deficiency in the etiology of preeclampsia is emerging (Garrido-Gómez et al., 2020). Here we found that EVT treated with CM from hESF decidualized in the presence of prednisolone had reduced *PGF* production. Serum PGF is reduced in early pregnancy serum of women who develop PE and is a biomarker used in the 1st trimester screening test for preterm preeclampsia (Akolekar et al., 2013). In this study hESF also had altered production of factors previously identified to be increased in the decidua of women with preeclampsia, including COL4A1 (Yong et al., 2014, 2015), LNPEP (Yong et al., 2014), TM9SF2 (Garrido-Gomez et al., 2017) and COTL1 (Garrido-Gomez et al., 2017). The impact of prednisolone treatment on the decidua and decidual function could be a novel mechanism by which prednisolone or other corticosteroid use increases preeclampsia risk.

Overall, this study demonstrates that prednisolone alters decidualized hESF and altered decidual-trophoblast interactions. The clinical consequences of these changes are unknown, however, as all available data suggests that corticosteroid administration has no beneficial effect for IVF (Kaye et al., 2017; Mohammadi Yeganeh et al., 2018), RPL (Tang et al., 2013; Cooper et al., 2019; Woon et al., 2020), or repeated implantation failure (Siristatidis et al., 2018) and the emerging evidence that corticosteroid use during pregnancy may be associated with poor obstetrical outcomes (Boyd et al., 2015; Bandoli et al., 2017), off-label use of corticosteroids, in particular prednisolone, during the period of decidualization (secretory phase of the menstrual cycle and the 1st trimester), should be carefully considered.

## Data Availability Statement

The datasets presented in this study can be found in online repositories. The names of the repository/repositories and accession number(s) can be found in the article/Supplementary Material.

## Ethics Statement

The studies involving human participants were reviewed and approved by Royal Women’s Hospital Human Research Ethics Committee Monash Health Human Research Ethics Committee. The patients/participants provided their written informed consent to participate in this study.

## Author Contributions

EG, EM, and ED wrote the main manuscript text. EG prepared Figures 1–4 and Supplementary Tables 2–4. TS prepared Figures 2–4 and Supplementary Tables 2, 3. SV and NW prepared Figure 3 and Supplementary Tables 2, 3. EM prepared Figures 1–4. All authors reviewed the final manuscript.

## Conflict of Interest

The authors declare that the research was conducted in the absence of any commercial or financial relationships that could be construed as a potential conflict of interest.

## Footnotes

1 https://david.ncifcrf.gov/
