# Supplementary material for: Prednisolone Alters Endometrial Decidual Cells and Affects Decidual-Trophoblast Interactions
Source: Front Cell Dev Biol. 2021 Apr 9;9:647496. doi: 10.3389/fcell.2021.647496 (PMC8063028; doi:10.3389/fcell.2021.647496)
Supplement: Supplementary file 1 [file Table_1.docx]

Supplementary Material

**Supplemental Table S1.** Primer sequences.

| **Gene of interest** | **Forward sequence 5`-3`** | **Reverse sequence 5`-3`** |
| --- | --- | --- |
| ARF6 | GGAAACTTGAAACCCTCATG | ACATCTCACCTGCAACATTC |
| BCAR1 | GACTCGCCAGATGGGCAGTA | TGTTCCAGTCGTTCAAACTGCTT |
| BMP2 | ACCCGCTGTCTTCTAGCGT | TTTCAGGCCGAACATGCTGAG |
| BMP7 | TCGGCACCCATGTTCATGC | ACGGCCTTGTAGGGGTAGG |
| ENG | TTTGTCTTCGGCAGTGCTTACT | TTTTCCGCTGTGGTGATGA |
| FLT1 | CGTAGAGATGTACAGTGAAA | GGTGTGCTTATTTGGACATC |
| GR | CCCTACCCTGGTGTCACTGT | GGTCATTTGGTCATCCAGGT |
| HOXA10 | GCAAAGAGTGGTCGGAAGAA | GATCCGGTTTTCTCGATTCA |
| IGFBP1 | TCACAGCAGACAGTGTGAGAC | CCCAGGGATCCTCTTCCCAT |
| IL6 | TACCCCCAGGAGAAGATTCC | TTTCAGCCATCTTTGGAAGG |
| IL10 | CTGCCTAACATGCTTCGAGA | TGGGTCTTGGTTCTCAGCTT |
| IL11 | GTGGCCAGATACAGCTGTCGC | GGTAGGACAGTAGGTCCGCTC |
| IL18 | GCATCAACTTTGTGGCAATG | TCCGGGGTGCATTATCTCTA |
| INHBA | CGAGGAAGTGGGCTTAAAGGG | TCACAGGCAATCCGAACGTCC |
| ITGB1 | ATTCCCTTTCCTCAGAAGTC | TTTTCTTCCATTTTCCCCTG |
| LIF | TGAACCAGATCAGGAGCCAACT | CCACATAGCTTGTCCAGGTTGTT |
| LIFR | AAGTTTATCCCCATACTCCTAC | CCTGGTAAATGCCAAGAAAG |
| MET | CAACCCGAATACTGCCCAGA | CCGGGACACCAGTTCAGAAA |
| PGF | CCCTTGGGTCTCCTCCTTTC | TGCTGCGGCGATGAGAATC |
| PLCG1 | TGTCCCACAGACCAACGC | ATTCCGCTTCCGCACCAG |
| PRL | TGACCCTTCGAGACCTGTTTG | CTTGCTCCTTGTCTTCGGG |
| sFLT1-e15a | CTCCTGCGAAACCTCAGTG | GACGATGGTGACGTTGATGT |
| STAT3 | TGGCCCAATGGAATCAGCTAC | CTGCTGGTCAATCTCTCCCA |
| VEGFA | GGCAGAATCATCACGAAGT | CACAGGATGGCTTGAAGAT |
