# Supplementary material for: Prednisolone Alters Endometrial Decidual Cells and Affects Decidual-Trophoblast Interactions
Source: Front Cell Dev Biol. 2021 Apr 9;9:647496. doi: 10.3389/fcell.2021.647496 (PMC8063028; doi:10.3389/fcell.2021.647496)
Supplement: Supplementary file 4 [file Data_Sheet_1.docx]

Supplementary Material

**Supplementary Methods.**

Mass spectrometry

Decidualized hESF cellular proteins following treatment with 0.5ug/ml prednisolone or vehicle control were identified using mass spectrometry. Day 14 cells were lysed and homogenized in ice-cold universal lysis buffer as previously described(Menkhorst et al., 2012).

*Sample Preparation:* 3ug total cellular protein quantified using BCA assay (Pierce) was used for Solid-Phase Protein Preparation as previously described(Dagley et al., 2019;Hughes et al., 2019). Briefly, SP3 protocol was carried with 10μg of extracted protein samples in a total volume of 50μL Triethyl ammonium bicarbonate buffer (TEAB) followed by reduction with 10 mM TCEP for 45 minutes at 37 °C and alkylation with 55mM Iodoacetamide for 45 minutes at 37°C in dark. Magnetic beads were prepared by combining 20μL of both, Sera-Mag Speed Beads A and B (GE Healthcare cat. no. 45152105050250; cat. no. 65152105050250) and washed two times with 200μL ddH2O and were re-suspend in 40μL ddH2O for a final working concentration of 50μg/μL. 2μL of pre-washed magnetic beads as well as 50μL 100% ethanol were added to each sample. Protein binding to the beads was facilitated in ThermoMixer at 24°C for 5 min at 1,000 r.p.m. After the binding is complete, tubes were placed in a magnetic rack and were incubated until the beads have migrated to the tube wall. The supernatant was removed and beads were washed thrice with 180μL of 80% ethanol. Beads were resuspended in 100μL of 100 mM TEAB and sonicated for 5 minutes in a water bath. The samples were then kept for overnight digestion at 37°C and 1000 rpm in a table-top thermomixer after adding sequencing-grade trypsin in an enzyme:protein ratio of 1:10. Upon digestion, peptides were recovered by collecting the supernatant. These peptides were lyophilized and stored until mass analysis.

*LC-MS/MS analysis:* Lastly for subsequent LC-MS/MS analysis, samples were reconstituted in 30 μL 2% acetonitrile:0.1% trifluoroacetic acid and were analysed on a LTQ Orbitrap Elite (Thermo Scientific) coupled to an Ultimate 3000 RSLC nanosystem (Dionex). The nanoLC system was equipped with an Acclaim Pepmap nano-trap column and an Acclaim Pepmap analytical column. 6μl of the peptide mix was loaded onto the trap column at 3% CH3CN containing 0.1% formic acid for 5 min before the enrichment column is switched in-line with the analytical column. The LC gradient used was 3% B to 20% B for 95 min, 20% B to 40% B in 10 min, 40% B to 80% B in 5 min and maintained at 80% B for the final 5 min before equilibration for 10 min at 3% B prior to the next analysis. The LTQ Orbitrap Elite mass spectrometer was operated in the data-dependent mode, spectra acquired first in positive mode at 240k resolution followed by collision induced dissociation (CID) fragmentation. Twenty of the most intense peptide ions with charge states ≥2 were isolated and fragmented using normalized collision energy of 35 and activation Q of 0.25 (CID).

*Data Analysis:* Raw data files were searched against the Human protein reference proteomes (UniProt Proteome ID: [UP000005640](https://www.uniprot.org/proteomes/UP000005640)) using MaxQuant-Andromeda (version 1.6.7.0). The false discovery rate (FDR) was set at 0.01 for both peptides and proteins. Search parameters were set as follows: variable modifications: Oxidation (M), Acetyl (Protein N-term); fixed modifications: cysteine carbamidomethylation. The analysis of the samples was based on the label-free quantification (LFQ) intensities. Initial analyses and visualization of proteomics data was performed using LFQ-Analyst (Figure 2A-C)(Shah et al., 2020). The data was statistically evaluated using Perseus software (version 1.6.7.0). The protein data was filtered categorically by row for reverse identifications (false positives), contaminants, and proteins “only identified by site”. The fold changes in the protein levels were evaluated by comparing the mean LFQ intensities amid all experimental groups. A protein was considered to be differentially expressed if the difference was statistically significant (p < 0.05), the fold change >1.5 and < 0.66 was identified with a minimum of 2 peptides. The mass spectrometry proteomics data have been deposited to the ProteomeXchange Consortium via the PRIDE(Perez-Riverol et al., 2019) partner repository with the dataset identifier PXD020543.

Assessment of protein function enrichment was performed using DAVID Bioinformatics Resources 6.8 (<https://david.ncifcrf.gov/>)(Huang da et al., 2009b;a), selecting *Homo sapiens* as the reference species.

**Supplemental Table S1.** Primer sequences.

**Supplemental Table S2.** hESF proteins significantly regulated by prednisolone treatment during decidualization.

**Supplemental Table S3.** hESF protein function enrichment following prednisolone treatment during decidualization.

**Supplemental Table S4.** Cell motility Array: pooled (n=2) isolated extravillous trophoblast gene expression.

Dagley, L.F., Infusini, G., Larsen, R.H., Sandow, J.J., and Webb, A.I. (2019). Universal Solid-Phase Protein Preparation (USP3) for Bottom-up and Top-down Proteomics. *Journal of Proteome Research* 18**,** 2915-2924.

Huang Da, W., Sherman, B.T., and Lempicki, R.A. (2009a). Bioinformatics enrichment tools: paths toward the comprehensive functional analysis of large gene lists. *Nucleic Acids Res* 37**,** 1-13.

Huang Da, W., Sherman, B.T., and Lempicki, R.A. (2009b). Systematic and integrative analysis of large gene lists using DAVID bioinformatics resources. *Nat Protoc* 4**,** 44-57.

Hughes, C.S., Moggridge, S., Müller, T., Sorensen, P.H., Morin, G.B., and Krijgsveld, J. (2019). Single-pot, solid-phase-enhanced sample preparation for proteomics experiments. *Nature Protocols* 14**,** 68-85.

Menkhorst, E.M., Lane, N., Winship, A., Li, P., Yap, J., Meehan, K., Rainczuk, A., Stephens, A.N., and Dimitriadis, E. (2012). Decidual-secreted factors alter invasive trophoblast membrane and secreted proteins implying a role for decidual cell regulation of placentation. *PLoS ONE* 7**,** e31418.

Perez-Riverol, Y., Csordas, A., Bai, J., Bernal-Llinares, M., Hewapathirana, S., D.J, K., Inuganti, A., Griss, J., Mayer, G., Eisenacher, M., Pérez, E., Uszkoreit, J., Pfeuffer, J., Sachsenberg, T., Yilmaz, S., Tiwary, S., Cox, J., Audain, E., Walzer, M., Jarnuczak, A.F., Ternent, T., Brazma, A., and Vizcaíno, J.A. (2019). The PRIDE database and related tools and resources in 2019: improving support for quantification data. *Nucleic Acids Res* 47**,** D442-D450.

Shah, A.D., Goode, R.J.A., Huang, C., Powell, D.R., and Schittenhelm, R.B. (2020). LFQ-Analyst: An Easy-To-Use Interactive Web Platform To Analyze and Visualize Label-Free Proteomics Data Preprocessed with MaxQuant. *Journal of Proteome Research* 19**,** 204-211.
